# Deep Brain Stimulation: Overview and Applications in the Context of Neuropsychiatric Conditions

**DOI:** 10.7759/cureus.93661

**Published:** 2025-10-01

**Authors:** Deirdre L Richardson, Eric Whitney

**Affiliations:** 1 Neurology, Western University of Health Sciences, Chino Hills, USA; 2 Neurosurgery, Arrowhead Regional Medical Center, Colton, USA

**Keywords:** adaptive dbs, autism spectrum disorder, connectomics, deep brain stimulation, dementia, neuromodulation, obsessive-compulsive disorder, treatment-resistant depression

## Abstract

Deep brain stimulation (DBS) is a neuromodulatory therapy involving the implantation of electrodes into brain structures in order to help normalize brain activity in a variety of neurological disorders. The mechanisms of DBS operate at micro-, meso-, and macroscale levels to influence neuronal signaling, synaptic reorganization, and network-wide connectivity between brain regions. Recent advances in connectomics and sensing technologies have allowed for more precise and adaptive stimulation strategies, increasing the potential to target complex, heterogeneous neuropsychiatric conditions. DBS has already been well-explored as a treatment for obsessive-compulsive disorder. Emerging research has explored the use of DBS for other neuropsychiatric conditions as well, including autism spectrum disorder (ASD), treatment-refractory depression (TRD), and dementia associated with Alzheimer’s disease (AD).

DBS for ASD shows promise in reducing self-injurious behaviors and aggression by targeting the nucleus accumbens (NAc), amygdala, and posteromedial hypothalamus. In TRD, DBS to the subcallosal cingulate gyrus (SCG), medial forebrain bundle (MFB), and ventral capsule/ventral striatum (VC/VS) has demonstrated significant antidepressant effects. For dementia and AD, DBS targeting the fornix and nucleus basalis of Meynert (NBM) has shown promise in slowing cognitive decline. Despite the variety of targets, connectomic analyses reveal overlapping cortical-subcortical network dysfunctions across these disorders. These findings offer insight into shared neurobiological mechanisms of these disorders, as well as guide refinement of therapeutic targets for future study.

Overall, DBS for neuropsychiatric conditions remains in its early stages, hindered by disorder heterogeneity and challenges in identifying optimal brain targets. Advances in functional neuroimaging, closed-loop stimulation, and machine learning-driven connectomic approaches can aid in target selection as well as better understanding the neuroanatomy and physiology underlying these complex conditions, which, in turn, lead to improved patient outcomes. Further research is necessary to establish standardized protocols and expand the therapeutic applications of DBS in neuropsychiatry.

## Introduction and background

Deep brain stimulation (DBS) is a form of neuromodulation that involves the surgical implantation of electrodes into deep brain structures [1]. The electrodes are traditionally wired to an implantable pulse generator (IPG) on the patient’s chest, not unlike a pacemaker. Upon proper lead placement, the stimulation settings are programmed, monitored, and adjusted over multiple outpatient appointments. DBS parameters that may be adjusted include stimulus amplitude, pulse width, frequency, and polarity of electrode contact(s) used [2]. 

DBS was initially introduced as a treatment for movement disorders in the 1980s, targeting the ventral intermediate nucleus of the thalamus (VIM) to treat severe tremor in Parkinson's disease (PD). Since then, DBS has achieved FDA approval for essential tremor (ET) and advanced PD in 1997, medication-refractory epilepsy, and gained FDA Humanitarian Device Exemptions for dystonia and OCD in 2003 and 2009, respectively. Brain regions that have been targeted include the subthalamic nucleus (STN), ventral intermediate nucleus (VIM), the internal part of the globus pallidum (GPi), the anterior nucleus of the thalamus (ANT), and the crus anterior of the internal capsule [3].

DBS effects depend on multiple factors: brain region, type of target tissue (gray vs white matter), connections to other brain regions, and properties of individually engaged axons and synapses, to name a few [4]. DBS is thought to exert its effects through micro-, meso-, and macro-level mechanisms. At the microscale, DBS alters neuroplasticity through stimulation of neurogenesis and synaptogenesis within the brain. Mesoscale structures refer to neural circuits. DBS stimulates both downstream (orthodromic) and upstream (antidromic) neuronal pathways, reorganizing functional connectivity between entire regions. On a macroscale, DBS influences whole-brain connectivity, promoting changes far beyond just the region being stimulated. For instance, DBS suppresses abnormal beta oscillations in PD to alleviate motor symptoms, both within the target regions (STN or GPi), as well as in distant cortical areas in the brain [5]. 

Additionally, DBS has anti-inflammatory and neuroprotective properties by decreasing levels of inflammatory cytokines, including interleukin-1 beta (IL-1β), interleukin-6 (IL-6), and tumor necrosis factor-alpha (TNF-α), thereby contributing to reduced chronic neuroinflammation. Electrotaxis, or the movement of cells in response to electric fields, also occurs around the electrodes. For instance, DBS stimulation was found to promote the shift of microglia from a pro-inflammatory (M1) to an anti-inflammatory (M2) state [1,6].

Precise placement of DBS leads in the brain is critical to achieving a successful outcome. Most preoperative appointments for DBS implantation focus on identifying the optimal brain target for the desired clinical effect. Historically, detailed images of the brain are laid over patient imaging as a guide, known as indirect targeting [7]. Later, the use of microelectrode neurophysiologic recordings (MERs) to look at electrical activity in the brains of patients during awake surgery allowed even better visualization in real time [2].

Connectomic DBS has also emerged as a groundbreaking approach, utilizing diffusion tensor imaging (DTI) and functional MRI (fMRI) to create a *map* of the brain’s structures and neural connections. By integrating connectomics with machine learning models, researchers are attempting to predict optimal DBS targets in a variety of neurologic and psychiatric conditions, giving the most tailored treatment possible [8].

Advances in sensing technologies allow us to detect neural signals in the form of local field potentials (LFPs), which act as biomarkers of brain activity. This has allowed for the development of closed-loop DBS systems, such as adaptive DBS (aDBS), which can adjust neuromodulation settings based on real-time feedback, tailoring stimulation to that specific patient without the need for an additional visit to an outpatient. Though aDBS is still in early stages, the technology shows promise in delivering tailored care [2,9,10]. 

DBS has already been approved for a variety of neurologic disorders, but other than OCD, DBS for neuropsychiatric conditions is still an emerging area of study. This is in part due to the challenge in identifying optimal targets for DBS. These conditions are heterogeneous, with complex symptomatology affecting multiple brain regions as well as diverse clinical presentations. A better understanding of these underlying neural circuits is crucial for the identification of biomarkers. For instance, overactivity within the corticostriatal thalamocortical (CSTC) has been identified as a hallmark biomarker for OCD symptomatology. 

The purpose of this review is to provide an overview of DBS outcomes in neuropsychiatric conditions, with special attention to pathophysiology and underlying neural circuitry. Conditions of focus will include obsessive-compulsive disorder, autism spectrum disorder (ASD), treatment-resistant depression, and cognitive disorders such as Alzheimer’s.

Methodology

A comprehensive search strategy was conducted using both PubMed and Google Scholar databases. Search terms included “Deep Brain Stimulation,” “DBS mechanism,” “DBS self-injury,” “DBS OCD,” “deep brain stimulation autism,” “DBS adaptive,” “DBS neuropsychiatric conditions,” “DBS dementia,” and “DBS Alzheimer’s.” Articles were eligible to be included if they were peer-reviewed and published in English. Studies not published in English were excluded. Both primary research studies and review articles were considered for inclusion if they addressed mechanisms or applications of DBS in neuropsychiatric conditions. The search covered publications through January 2025.

## Review

Obsessive-compulsive disorder

Obsessive-compulsive disorder (OCD) is a mental health condition characterized by persistent, intrusive, and unwanted thoughts (obsessions) that give the individual great anxiety, and repetitive behaviors (compulsions) that act to temporarily relieve the anxiety. OCD can be debilitating, significantly impacting quality of life. Treatment traditionally involves a combination of psychotherapy and medications such as SSRIs, TCAs, or anti-psychotics. However, 40-60% of cases prove to be refractory to conventional treatments, leading to surgical intervention and neuromodulation approaches [11].

The pathophysiology of OCD, in particular, is thought to involve abnormalities within the CSTC loop [12]. The CSTC loop connects parts of the cortex, striatum, and thalamus via the internal capsule for goal-directed and habit-forming behaviors and emotional regulation. The anterior limb of the internal capsule (ALIC) has been particularly implicated in OCD; it is a white matter tract that connects the orbitofrontal cortex (OFC), anterior cingulate cortex (ACC), and dorsolateral prefrontal cortex (dlPFC), with more subcortical structures such as the ventral portion of the striatum, also known as the nucleus accumbens (NAc). Hyperactivity of key regions of the CSTC, such as the OFC, ACC, and striatum, has been implicated in OCD [13,14].

The cortex remains our primary center of reasoning and decision-making. Cortical areas such as OFC and dlPFC help manage reward value and subjective pleasantness, emotional regulation, sensory integration, learning, and decision-making [15]. Within the striatum, the NAc projects to the ventral tegmental area (VTA) within the mesolimbic dopamine pathway to modulate motivation and goal-directed behaviors and the brain's reward system [16].

Target brain regions for OCD DBS include the FDA-approved ALIC, as well as the NAc, subthalamic nucleus (STN), and the ventral capsule/ventral striatum (VC/VS) [17]. DBS is thought to modulate the synchrony of oscillatory activities within the CSTC loop, particularly gamma and theta rhythms, to reduce circuit hyperactivity, which can, in turn, quell OCD symptom severity [18]. At the level of the striatum and NAc, DBS curbs abnormal dopamine activity, which reduces the reward sensitivity associated with compulsive behaviors [19].

OCD DBS is effective, having received FDA HDE in 2009. A meta-analysis of 25 studies conducted from 2003 to 2020 showed DBS significantly improved symptoms in TR-OCD, with an average 50% reduction in OCD symptoms and two-thirds of patients achieving full response [20]. Their study further shows significant improvements not just in obsessive-compulsive symptoms, but in depression and anxiety, as well as improved overall global functioning following DBS, which have been replicated in real-world studies [20,21]. Connectomic analyses suggest that these successful outcomes in OCD DBS targeted to either the ALIC or STN correlate with greater connectivity to cortical regions, including the ACC, insula, and precuneus [22]. There is an overlap between OCD and many other neuropsychiatric conditions in symptomatology and implicated brain regions. Dysregulation in the NAc, for example, has been linked to the repetitive behaviors in ASD, not unlike its link to the repetitive compulsions of OCD [19]. A better understanding of these targets and outcomes could expand the approved use of DBS for other neuropsychiatric conditions. 

Autism spectrum disorder 

With success in the use of DBS for OCD, attention has been turned to other neuropsychiatric conditions, such as ASD. ASD is a group of neurodevelopmental disorders characterized by differences in social communication as well as restrictive and repetitive behaviors (RRBs) that can severely impact a person’s quality of life. Some autistic people also exhibit severe aggressive or self-injurious behaviors. These often stem from an inability to cope with overwhelming sensory or emotional stimuli, but remain extremely distressing to the individual, as well as their families. Applied behavior analysis (ABA) is one of the mainstay therapies for ASD, focusing on improving social skills and reducing problematic behaviors that could be harmful to the patient and their functioning; severe behaviors may be managed with medications such as antipsychotics, but aren't effective in every case [23]. 

The application of DBS for ASD is still relatively recent. Early case studies emerged in the 2010s, which have focused on these severe behaviors that proved refractory to medications. Commonly targeted regions included the NAc, BLA, ALIC, VS/VC, and posteromedial hypothalamus (pHyp) [24].

DBS to the ALIC, NAc, BLA, and pHyp was found to improve severe aggression and SIBs in patients with ASD and multiple comorbidities who were refractory to pharmacologic intervention [24]. Another study reported significant reductions in aggressiveness and self-injury in five autistic patients following DBS stimulation of the bilateral pHyp, improving functionality and social adaptation [25]. pHyp affects sensory processing in the thalamocortical circuit, implying that DBS may reduce excessive sensory input that may be overwhelming to the individual [26]. Currently, a prospective pilot trial is underway to assess the safety and efficacy of NAc-DBS in children with ASD and severe SIBs [27]. 

In the autistic brain, differences in social communication are linked to altered processing in higher-order cognitive networks (including disruptions in the default mode network (DMN) and salience networks), while RRBs and sensory disturbances are associated with disruptions in subcortical and thalamocortical connections [26]. Implicated subcortical areas include the caudate, amygdala, and NAc [26,28]. 

Overlap has been discovered between circuits engaged in OCD and ASD. A connectomic analysis identified nine patients with ASD and multiple comorbidities who were receiving DBS for aggression or SIBs to the NAc, GPi, ALIC, pHyp, VC/VS, and BLA. They were able to demonstrate common engaged neural circuitry within the ALIC for patients who received DBS to the NAc, VC/VS, or pHyp. These *hot spots* closely overlap with those identified in OCD [22]. Considering the behaviors targeted were aggression and SIB, behaviors which are nonspecific to ASD, this *hot spot* could be a region worth studying for other neuropsychiatric conditions involving emotional and behavioral dysregulation, beyond just OCD and ASD.

ASD DBS for differences in social communication hasn’t been explored in humans, although there could be utility in looking at the underlying neurophysiology of the implicated DMN as well. The DMN is a collection of many interconnected cortical regions (including dlPFC, anterior PFC, and medial temporal lobe) that are active when the individual is not preoccupied with external stimuli (i.e., the individual’s *default* internally focused thought processes). The DMN also has connections with the amygdala, striatum (including ALIC), and NAc (among other regions), making it important for self-referential thought, social cognition, planned behaviors, and memory [29]. DBS in these regions may be explored with the therapeutic aim to better support things like sensory-processing and self-regulation during social interactions in individuals with ASD. There is also a functional and connectivity overlap between the DMN and CSTC loops. Given DBS’s ability to affect tissue beyond the targeted local region, and its potential to aid emotional and behavioral regulation in OCD and ASD, a closer examination of these interconnected regions is warranted.

Treatment-refractory depression 

DBS has also been explored as a therapeutic option for depression. Depression, clinically referred to as major depressive disorder (MDD), is a debilitating mental health disorder characterized by persistent low mood and anhedonia, as well as a myriad of other cognitive or physical symptoms that vary person to person. The etiology of MDD is complex, related to a combination of genes, environment, and neurobiological factors. The monoamine hypothesis states that depression is based on depletion of the neurotransmitters serotonin (5-HT), norepinephrine (NE), or dopamine (DA). Medications targeting these neurotransmitters, such as selective serotonin reuptake inhibitors (SSRIs), are effective in most, but approximately 20-30% will go on to experience treatment-refractory depression (TRD), a lack of response to 2+ trials of antidepressants [30-32]. 

With regards to TRD, it is important to note that less invasive and more accessible neuromodulation techniques, such as transcranial magnetic stimulation (TMS) and vagus nerve stimulation (VNS), have demonstrated efficacy in managing TRD [33]. DBS is a comparatively more invasive option, but it could still have utility for cases refractory to other forms of neuromodulation, especially due to its capabilities for more specific targeting.

Generally, hyperactivity of neurons in cortical regions (OFC, dlPFC, ACC, and subcallosal cingulate gyrus (SGC)), as well as abnormal connectivity to the striatum and amygdala, have been implicated in MDD [34]. Volume reductions have been noted in the PFC (including OFC and ACC), hippocampus, and amygdala [35,36]. Thus, DBS targets studied for TRD include the SCG, medial forebrain bundle (MFB), ALIC, NAc, inferior thalamic peduncle (ITP), and lateral habenula (LH). 

Overall, TRD DBS is favorable. DBS to the SGC, VC/VS, MFB, and NAc was found to significantly reduce depressive symptoms in TRD in a meta-analysis across 14 studies [37]. SCG-DBS is particularly efficacious, shown to significantly decrease depression symptoms at follow-ups from 12 months up to 11 years [38,39]. In looking closer at specific regions studied, DBS to the ALIC was found to normalize right amygdala responsiveness and connectivity to other cortical and sensorimotor regions [40]. NAc-DBS also improved depression and anxiety symptoms, with short-term stimulation causing a decrease in D2 binding in the amygdala, caudate, and substantia nigra [41]. 

The brain regions and neural circuits implicated in TRD have a strong overlap with those implicated in both OCD and ASD. Connectomics have found overlapping regions of connectivity from areas targeted for OCD- and TRD-DBS, namely, the OFC [42]. The DMN is also implicated in TRD. In a connectomic analysis of TRD DBS targets, the SCG, MFB, ALIC, NAc, ITP, and LH were found to have common connectivity to the anterior PFC region known as Brodmann Area 10 (BA 10) as well as the amygdala, suggesting these could be “hubs” central to the neural circuit of TRD [43]. BA 10, a major component of the DMN, is known to be involved in a wide variety of functions, including decision-making, reward and conflict processing, memory, behavior, and even pain [44]. TRD is associated with hyperactivity of brain regions within the DMN, but a reduced functional connectivity between those and other brain areas (by interfering with both glutamatergic and GABAergic neurons) [45]. Further exploration of these complex cortical-subcortical connections is critical to developing personalized and adaptive DBS protocols for patients with TRD. 

Dementia and Alzheimer's disease 

Dementia is a general term that describes symptoms or illnesses that affect cognitive functions such as memory, attention, and problem-solving. Alzheimer’s disease (AD) is the most common form, accounting for ~60% of cases [46]. AD is neurodegenerative, meaning it worsens over time, proving to be devastating to patients and their families. The progressive cognitive decline is most associated with the accumulation of amyloid-beta (Aꞵ) plaques within regions of the brain, including the hippocampus and other cortical regions [47]. Also commonly implicated in AD is the nucleus basalis of Meynert (NBM), a region that provides cholinergic input to the cortex, hippocampus, and cingulate gyrus. Acetylcholine (ACh) is imperative for attention, arousal, and memory functions in the brain, and degeneration of the NBM is considered a hallmark of AD, leading to low ACh levels and impacted cognition [48]. 

Many current treatments for AD have focused on targeting the accumulation of Aꞵ plaques within the brain, based on the identification of biomarkers in the cerebrospinal fluid (CSF) or blood (including Aꞵ-42, tau, and others). Anti-amyloid monoclonal antibodies, which target Aꞵ, are continuously being developed and studied, which remarkably slow disease progression, but these therapies ultimately do not stop or reverse cognitive decline [49]. 

On the level of neural circuits, memory problems in AD have been strongly linked to dysfunction within the Papez circuit (PC). The PC connects cortical structures to limbic ones; it comprises the hippocampus, parahippocampal gyrus (PHG), anterior thalamic nuclei (ATN), cingulate gyrus, fornix, and entorhinal cortex (EC) [50]. Reductions in grey-matter volume have been found within many of these areas [50]. The DMN is also impacted in dementia and AD, given its connections to the PC. DMN neurons have been shown to have reduced connectivity to brain regions such as the hippocampus in people with dementia/AD [51]. In the time course of dementia, the hippocampus, amygdala, and EC have been noted to be impacted earlier than other brain regions [52]. The capability of DBS to target underlying neural circuits that may be responsible for cognitive decline makes it worth further investigation. 

DBS is only newly emerging as an option for AD to slow the rate of cognitive decline. DBS targets include the NBM as well as a key PC node, the fornix, and results so far are promising. A systematic review of 14 studies noted a slowdown in cognitive decline based on psychometric scales used in cognitive decline, such as the Clinical Dementia Rating Scale Sum of Boxes score, Mini-Mental State Examination, and Alzheimer’s Disease Assessment Scale [53]. DBS to the NBM or fornix was found to normalize aberrant signaling within the PC, with associated activation of neurons in the hippocampus as well as the DMN, leading to slower declines in cognition over time [50]. NBM-DBS in particular was found to increase connectivity between the parietal cortex and PHG in the short term after DBS was initiated [54].

Future directions

Future research in DBS for neuropsychiatric conditions may benefit from a shift from purely target-centric approaches toward network- and state-based models of intervention. Patient-specific connectomic mapping and machine learning-assisted analyses hold promise for refining lead placement and predicting outcomes across heterogeneous neuropsychiatric disorders such as OCD, ASD, TRD, and dementia. Future work in DBS should also expand beyond our current targets. In ASD, for example, DBS applications could move beyond the treatment of aggression and self-injury and towards addressing disrupted social-communication networks, with the potential to support improved self-regulation for the individual in social settings. Dementia research may benefit from pairing stimulation of memory circuits with task-specific or phase-locked paradigms to potentially further enhance neuroplasticity and slow cognitive decline.

On a broader level, future trials should prioritize the development and use of DBS based on biomarkers that are grounded in underlying neural circuit dynamics for each patient. Studies capturing long-term safety, potential adverse effects, and patient-reported outcomes are equally critical, particularly for more vulnerable patient populations who may have impaired decision-making capacity. Finally, evolving hardware innovations, including directional leads, rechargeable pulse generators, and remote programming, are another big step towards improving both treatment precision and accessibility for our patients. Taken together, these directions point toward a future where DBS evolves into a more adaptive and equitable intervention for patients with complex neuropsychiatric disorders.

## Conclusions

Neuropsychiatric disorders are inherently complex, with diverse underlying mechanisms in the brain that can vary widely in how they manifest clinically. DBS works via micro-, meso-, and macro-scale mechanisms to exert its therapeutic effect. Precise lead placement is extremely important for a good clinical outcome, but requires a thorough understanding of the neural circuits underlying the disorder and its symptoms. OCD DBS is effective, receiving its FDA HDE in 2009, but research is still currently underway in other disorders, including TRD, ASD, and dementia/AD. Although these conditions are each complex and multifaceted in their own right, they share some interesting overlapping network dysfunctions related to cortical-subcortical signaling. Further research into the different neural circuits underlying these disorders, particularly through advanced connectomic approaches, is essential. A better understanding of neuroanatomy, physiology, and the variability of individual brain networks will ultimately be what informs more personalized and effective DBS interventions.
